# Editorial: Pediatric preventable diseases

**DOI:** 10.3389/fped.2023.1187008

**Published:** 2023-03-31

**Authors:** Elena Bozzola, Alberto Eugenio Tozzi

**Affiliations:** ^1^ Pediatric Unit, Bambino Gesù Children's Hospital, IRCCS, Rome, Italy; ^2^ Predictive and Preventive Medicine Research Unit, Bambino Gesù Children's Hospital, IRCCS, Rome, Italy

**Keywords:** prevention, preventale diseases, children, health, immunization, education

**Editorial on the Research Topic** Pediatric preventable diseases

Preventable diseases are a leading cause of disability and death worldwide and prevention remains the most efficacious medical intervention, even though its effect may not be immediately apparent. The advancement of research and technology plays a crucial role in reducing child mortality from preventable diseases, leading also to improved neurophysiological development for children and adolescents.

The World Health Organization advocates for national strategies to strengthen ongoing control of infectious diseases and reduce associated transmission, mortality, and morbidity. To develop appropriate immunization recommendations and implement public health policies, timely epidemiologic evaluations are crucial in understanding the circulation of infectious diseases and identifying high-risk populations in the pediatric age group. For instance, orphans and vulnerable children in African areas are a high-risk group for infectious diseases, including human immunodeficiency virus infection, as shown by socioeconomic analyses. Education of families also plays a major role: education of mothers on diarrhea management has been linked to reduced death rates (Shah et al.; Gessesse and Tarekegn).

Immunization is an effective approach to prevent the substantial impact of preventable infectious diseases. Through vaccination, smallpox has been eradicated, and the prevalence of preventable infectious diseases, such as measles, diphtheria, and whooping cough, has been significantly reduced worldwide. The implementation of immunization programs has also led to a decrease in hospitalization costs and socio-economic burden. Currently, new vaccines and immunization strategies are being developed and adapted to the epidemiological burden of preventable infectious diseases. For example, different immunization options have being developed to prevent respiratory syncytial virus (RSV) infections in children. Of note, evidence indicates that RSV is responsible for almost 13 million cases of lower respiratory tract infections, 2.2 million hospitalizations, and 66,300 deaths globally each year Bont et al.

Effective health messages, immunization strategies, and risk factor awareness require robust communication strategies. It is crucial to communicate the benefits of immunization strategies to parents using simple and appropriate language. Using communication methods such as social media can enhance the dissemination of health information, facilitate interactions, boost family confidence and compliance, and positively influence parental vaccination acceptance. These strategies can help reassure parents about the safety and efficacy of immunization strategies and counteract infodemics Di Mauro et al.

To enhance the quality of life of children and prevent disabilities in later life, high-quality research should focus on major modifiable risk factors for non-infectious preventable pediatric diseases such as tobacco use, unhealthy diets, and physical inactivity. Early detection of high-risk behaviors is essential to achieving this goal. For instance, identifying predictors of childhood obesity can prevent long-term consequences such as metabolic syndrome and diabetes Byeon.

Similarly, reducing pollution can decrease respiratory infections in children, preventing long-term sequelae such as asthma. Evidence indicates that short and long-term exposure to air pollutants, including PM2.5, may lead to respiratory diseases in children, regardless of country development status. Fine particulate's small size enables easy penetration of human barriers, causing tissue damage and lung inflammation Liu et al.

Screening guidelines should also be widely implemented to prevent diseases, such as amblyopia, a common neurodevelopment disorder among children Yan et al.

Secondary prevention is also essential to reduce the burden and prevent long-term consequences. For instance, the rising number of myopia cases in adolescents emphasizes the need for developing strategies to slow the progression of visual impairment and its consequences. Multifocal lenses have been proven an effective approach to controlling myopia progression, thereby avoiding complications such as macular degeneration, retinal detachment, glaucoma, and premature cataracts Chen et al.

Creating a safe environment through preventive measures is crucial in avoiding pediatric diseases and disabilities caused by unintentional injuries. The ingestion of foreign bodies is a common clinical problem, particularly in the youngest age group, aged 6 months to 3 years. These children may be unaware of the risks associated with pediatric foreign body ingestion, particularly in cases of accidental ingestion of magnets or chemicals Ding et al.

